# Ionized Jet Deposition of Calcium Phosphates-Based Nanocoatings: Tuning Coating Properties and Cell Behavior by Target Composition and Substrate Heating

**DOI:** 10.3390/nano13111758

**Published:** 2023-05-29

**Authors:** Matteo Montesissa, Giorgia Borciani, Katia Rubini, Francesco Valle, Marco Boi, Nicola Baldini, Elisa Boanini, Gabriela Graziani

**Affiliations:** 1 Department of Biomedical and Neuromotor Sciences, University of Bologna, 40138 Bologna, Italy; 2 Department of Chemistry “Giacomo Ciamician”, University of Bologna, 40126 Bologna, Italy; 3 Institute of Nanostructured Materials, National Research Council, 40129 Bologna, Italy; 4 BST Biomedical Science and Technologies and Nanobiotechnology Laboratory, IRCCS Istituto Ortopedico Rizzoli, 40136 Bologna, Italy

**Keywords:** coatings, orthopedics, dentistry, calcium phosphates, ionized jet deposition, hydroxyapatite, brushite, beta-tricalcium phosphate, biomimicry, cell adhesion

## Abstract

Calcium phosphate-based coatings are widely studied in orthopedics and dentistry for their similarity to the mineral component of bone and their capability to promote osseointegration. Different calcium phosphates have tunable properties that result in different behaviors in vitro, but the majority of studies focus only on hydroxyapatite. Here, different calcium phosphate-based nanostructured coatings are obtained by ionized jet deposition, starting with hydroxyapatite, brushite and beta-tricalcium phosphate targets. The properties of the coatings obtained from different precursors are systematically compared by assessing their composition, morphology, physical and mechanical properties, dissolution, and in vitro behavior. In addition, for the first time, depositions at high temperature are investigated for the further tuning of the coatings mechanical properties and stability. Results show that different phosphates can be deposited with good composition fidelity even if not in a crystalline phase. All coatings are nanostructured and non-cytotoxic and display variable surface roughness and wettability. Upon heating, higher adhesion and hydrophilicity are obtained as well as higher stability, resulting in better cell viability. Interestingly, different phosphates show very different in vitro behavior, with brushite being the most suitable for promoting cell viability and beta-tricalcium phosphate having a higher impact on cell morphology at the early timepoints.

## 1. Introduction

Calcium phosphate (CaP)-based ceramic coatings have been widely studied for applications in dental and orthopedic implants because of their intrinsic bioactivity and biocompatibility derived from their similarity to the mineral component of bone [1,2].

The aim of these coatings is to favor a firm bonding between otherwise bio-inert metallic implants and the surrounding host bone tissue [3] to avoid mobilizing the implant through stimulation of new bone formation. The latter occurs due to the progressive dissolution and remineralization of the coating, ultimately leading to bone formation [4]. Remineralization and bone formation depend on the coating’s composition and crystallinity and on the rate of ion release, which can be due both to the partial dissolution of the coating on the surface and to the ion exchange with calcium ions from the body fluids [5]. For this reason, thin films (thickness below 1 µm) having high adhesion to the substrate and a nanostructured surface texture are desired [1]. In addition, a tunable composition is required to tailor coating solubility; that is, the amount and type of ions released in the peri-implant environment, which determines the biological behavior of the coatings [1]. In particular, hydroxyapatite-based coatings (HA–Ca_10_(PO_4_)_6_(OH)_2_) have been widely explored with positive results for enhancing the biocompatibility of the substrate and the osseointegration of the implants [6]. Recently, much attention has moved to other calcium-phosphate compounds such as dicalcium phosphate dihydrate (DCPD–CaHPO_4_·2(H_2_O)) and beta-tricalcium phosphate (β-TCP–Ca_3_(PO_4_)_2_ because of their higher solubility and faster bone-formation rate. In particular, DCPD displays high reactivity in solution and can undergo hydrolysis to be converted into hydroxyapatite [7,8] However, deposition of DCPD by plasma-assisted techniques has never been reported, while deposition of β-TCP has been tested [9]. 

Together with composition and crystallinity, the morphology of biointerfaces is also of great importance for guiding cell behavior [10,11] as morphological cues at the micro- and nano-scale can affect cell adhesion, proliferation and even differentiation [12]. 

In previous studies, the authors demonstrated that nanostructured thin films having surface roughness and a composition perfectly resembling that of the deposition target can be achieved by ionized jet deposition (IJD) [13,14,15]. In addition, because of nanostructuring and biomimetic composition, the coatings can guide host-cell behavior by increasing early proliferation and determining their morphology, which later determines osteogenic differentiation [11]. Herein, IJD was applied to realize different calcium phosphate films, to obtain coatings with high adhesion, nanostructured texture and homogeneity on the substrate surface (thickness below 1 µm). IJD is a plasma-assisted technique in which the target material is ablated by means of a pulsed electron beam [14]. In the present study, IJD was selected over similar techniques since it allows the deposition of nanostructured thin films that have good control over composition. Indeed, it permits high fidelity in the stoichiometry transfer from the deposition target to the coating compared to alternative techniques, such as Plasma Spray that often provokes the deposition of a significant number of decomposition phases, and magnetron sputtering, which causes preferential sputtering for phosphorous, thereby affecting the composition of the calcium phosphates coatings [3,14,16,17]. In general, IJD permits deposition [18,19], but recent modifications permit it to perform at temperatures up to 400 °C for metallic prostheses applications. Because it occurs in a vacuum using hot plasma, the deposition of organic materials and/or onto hydrated materials cannot be performed. 

The possibility of tuning the morphology, composition and crystallinity of the films provided by IJD is crucial for use in tissue regeneration since all the above-mentioned parameters have a key role in influencing the biological behavior of the films. Crystallinity is a key parameter for coating stability and influences solubility, which in turn determines the number of ions released in the microenvironment, thus having important effects on cell behavior and communication. Indeed, the different ions released can influence cell adhesion and proliferation, bone metabolism, osteoblast and osteoclast activity and stimulate new bone formation [5]. At the same time, morphological features such as the micro- and nano-scale surface topography, are important for conditioning cell adhesion, proliferation and differentiation [20]. Therefore, we exploited this capability to conserve target stoichiometry to deposit different CaP phases: HA, β-TCP, and DCPD. We verified the feasibility of this approach and compared the characteristics of the different coatings. At the same time, we also implemented, for the first time, deposition at high temperature to regulate the solubility and adhesion of the different coatings. 

The composition of the coatings was investigated to study its correspondence to the deposition target and demonstrate the feasibility of depositing different phosphates. The surface morphology of the coatings was studied for each material since it also depends on target characteristics. Furthermore, the impact of thermal treatment during deposition was tested for its effect on (i) phase composition, (ii) crystallinity, (iii) surface morphology and nano-scale features, (iv) coating adhesion. Finally, we investigated the impact of different coatings on cell viability and morphology.

## 2. Materials and Methods

### 2.1. Materials 

HA, β-TCP and DCPD powders were prepared starting from Ca(NO_3_)_2_·4H_2_O, (NH_4_)_2_HPO_4_, NH_4_OH, Na_2_HPO_4_·12H_2_O, NaH_2_PO_4_·H_2_O, Ca(CH_3_COO)_2_·H_2_O, Ca(CH_3_COO)_2_·H_2_O, CH_3_COOH, CaCO_3_ (Carlo Erba, Milano, Italy). 

Medical-grade titanium alloy (Grade 23 Ti6Al4V ELI alloy, Citieffe S.r.l., Bologna, Italy) cylinders and plates (5/10 mm diameter, 3 mm thickness, and 20 × 20 × 5 mm^3^ size, respectively, all having a microstructured surface (Ra = 5 µm)), and silicon wafers (p-type doped monocrystalline (100) native silicon, size 5 × 5 mm^2^, thickness 1 mm, Fondazione Bruno Kessler, Trento, Italy) were used as deposition substrates. The titanium alloy cylinders had the same composition and surface texture of a standard orthopedic prosthesis.

### 2.2. Target Preparation and Characterization 

The synthesis of HA nanocrystals was performed under an N_2_ atmosphere using CO_2_-free distilled water: 50 mL of 1.08 M Ca(NO_3_)_2_·4H_2_O solution at pH adjusted to 10 with NH_4_OH was heated to 90 °C and 50 mL of 0.65 M (NH_4_)_2_HPO_4_ solution (pH 10 adjusted with NH_4_OH) was added dropwise under stirring. The resulting suspension was stirred for 5 h at 90 °C, after which the precipitate was isolated by centrifugation at 10,000× *g* rpm, repeatedly washed with CO_2_-free distilled water and dried at 37 °C.

DCPD crystals were synthesized using 600 mL of a solution containing 0.08 mol of Na_2_HPO_4_·12H_2_O and 0.08 mol of NaH_2_PO_4_·H_2_O, at a pH adjusted to 4 with glacial CH_3_COOH. The solution was heated at 37 °C and 200 mL containing 0.16 mol Ca(CH_3_COO)_2_·H_2_O was added dropwise over a period of about 60 min, under mild stirring. Afterwards, the precipitate was stored in contact with the mother solution for 10 min, filtered and then repeatedly washed with deionized water and dried at 37 °C.

β-TCP powder was obtained by the solid state reaction of a mixture of CaCO_3_ and CaHPO_4_·2H_2_O in a molar ratio of 1:2, at 1000 °C for 10 h. After cooling, the solid product was ground in a mortar.

Hydroxyapatite, beta-tricalcium phosphate and brushite targets (HA-t, β-TCP-t and DCPD-t) were prepared by pressing 4.75 g of powder (HA, β-TCP and DCPD) and 0.25 g of starch into cylindrical molds (Ø = 2.8 cm), thus obtaining disc-shaped samples.

Target composition was characterized by XRD (PANalytical X’Pert PRO powder diffractometer equipped with a fast X’Celerator detector (Malvern Panalytical, Milan, Italy)), CuKα radiation, 40 mA, 40 kV; step size of 0.1 2θ°, time/step 100 s and FT-IR (Perkin Elmer Spectrum two (Perkin Elmer Italia, Monza, Italy), diamond crystal, resolution 4 cm^−1^, accumulation 16 scans, step size 1 cm^−1^).

### 2.3. IJD Deposition 

HA, β-TCP and DCPD coatings (HA-c, β-TCP-c and DCPD-c) were realized by IJD (Noivion Srl, Rovereto (TN), Italy). The IJD technique uses physical vapor deposition (PVD) to exploit a high-frequency and high-energy pulsed electron beam to ablate the target material. An effect of the interaction of the electron beam with the target, was the generation of a plasma plume from the ionized target material, which was accelerated towards the surface of the selected substrate where the coating started to grow in globular aggregates [21]. For the deposition, all targets (HA-t, β-TCP-t and DCPD-t) were mounted on a rotating target holder and ablated by a fast pulse (100 ns) of high energy (10 J) and high-density (109 W cm^−2^) electrons. To ensure uniformity in deposition, the substrate kept rotating during the whole process. Target–substrate distance was set to 80 mm based on previous results [20,21,22]. The vacuum chamber was kept at a pressure of 2 × 10^−4^ mbar, and deposition was carried out in an oxygen flow. The working voltage and electron beam frequency were set to 18 kV and 7 Hz, respectively, and the deposition time was set to 30 min based on preliminary tests of CaP coating deposition [11,14,17]. To evaluate the effects of substrate temperature on phase formation, the deposition was carried out both at room temperature (RT) and at 400 °C (T400), the highest temperature permitted by the IJD system. In addition, previous results from post-treatment annealing [17] showed that heating the coatings at 400 °C increased coatings crystallinity and adhesion without leading to decomposition phases or cracking. Here, the substrate was heated prior to deposition for a time suitable for achieving the selected temperature and avoiding temperature fluctuations. Afterwards, the temperature was kept constant for the deposition. The effects of substrate temperature on coating composition, morphology and crystallinity were determined. All tests were performed on Ti6Al4V, except for the FT-IR and scratch tests, which were performed on silicon wafers and titanium slabs, respectively.

### 2.4. Film Characterization

Coating morphology and surface texture dependent on deposition temperature and material were evaluated by a Field Emission Gun Scanning Electron Microscope (FEG-SEM, Zeiss Leo-1530 equipped with InLens detector and operating at 1 kV (Zeiss, Milan, Italy)) equipped with an Energy Dispersive X-ray spectrometry system (EDS, Oxford Instruments equipped with a SDD detector (Abingdon-on-Thames, UK)) for the elemental composition analysis. The substrates had previously been made conductive by sputtering with gold. Starting from FEG-SEM images at 15,000× magnifications, the dimensions of the aggregates that formed the coating were measured by ImageJ software (National Institutes of Health, USA). To achieve this, 3 non-overlapping areas of two samples were selected and the maximum (Dm), minimum (dm) and average diameter (da) were calculated for each area and then averaged. In addition, the aggregate diameter distribution was analyzed by dividing the diameter obtained from five different groups: d < 100 nm, 100 nm ≤ d < 250 nm, 250 nm ≤ d < 500 nm, 500 nm ≤ d < 1000 nm and d ≥ 1000 nm. The percentage of aggregates having diameters within each range was calculated. 

The coating topography and roughness were studied by Atomic Force Microscopy (Multimode VIII AFM equipped with a Nanoscope V controller, Bruker Italia srl, Milan, Italy). The AFM was operated in the ScanAsyst Imaging mode, by regulating the maximum load, and by a NT-MDT cantilever (NSG10, nominal spring constant: 3.1 N/m). Surface roughness (Root Mean Square–RMS by Gwyddion) was calculated based on 2 × 2 μm^2^ AFM images, after performing a plane subtraction 0-order line-flattening. 

Coating composition was evaluated by FT-IR and EDS. FT-IR spectra were acquired from 16 scans in attenuated total reflectance (ATR) mode at a resolution of 4 cm^−1^ and a data interval of 1 cm^−1^. EDS analysis was performed as described above for the targets by selecting 3 non-overlapping regions of two different samples. The Ca/P molar ratio was analyzed for every type of coating and compared with the stoichiometric composition of the relative target.

Wettability was evaluated using a KSV CAM101 instrument under ambient conditions by recording the side profiles of deionized water drops for image analysis. The shape of the drop was recorded in a range of 0–10 s, by collecting an image every 0.1 s. At least 3 drops were observed for each sample.

Adhesion to the substrate was studied using a micro-scratch test following ISO 20502: 2016 (Micro-Scratch Tester, CSM Instruments—Anton Paar S.r.l, Peseux, Switzerland, equipped with a conical Rockwell C stylus with spherical apex indenter tip–angle 120° and sphere radius 100 μm). The analyses were performed on titanium alloy plates, averaging 5 different scratch tests for each condition. A distance of 0.5 mm was kept between measures to avoid any alteration in results due to previous test tracks. The tip was subjected to a progressive normal loading from 0.01 to 10 N moved across the surface of the coated samples with an indenter traverse speed of 10 mm/min and a loading rate of 10 N/min. Worn tracks were examined by a reflected-light microscope (magnification 20× and 50×), to determine the failure modes of the coatings and associate them with the critical normal load (Lc) at which they occurred. Three different behavior and Lc values were observed: Lc1 was associated with the initial detachment of the coating from the substrate; Lc2 with coating delamination or spallation (evident detachment of film fragments) and Lc3 corresponded to the complete penetration of the coating by the indenter tip; hence, coating failure.

XRD analyses were performed on coatings with PANalytical X’Pert PRO powder diffractometer equipped with a fast X’Celerator detector (CuKα radiation (Malvern Panalytical, Milan, Italy), 40 mA, 40 kV; step size of 0.1 2θ°, time/step 100 s). Further data collections in relevant 2θ° ranges were performed (step size of 0.08 2θ°; time/step 3000 s). 

### 2.5. Stability Profile

To analyze the stability of the 400 °C coatings, dissolution tests were performed on titanium alloy cylinders. Before the tests, the samples were sterilized for 1 h under UV light. Then, they were put in 24 multi-wells, submerged in 2 mL of Minimum Essential Medium Eagle Alpha Modification (α-MEM), and incubated at 37 °C with 95% humidity. The samples were kept in the medium for different experimental times: 1, 3, 7 and 14 days. At each timepoint, samples were collected, washed in distilled water and observed by FEG-SEM to observe the residual presence of the coatings.

### 2.6. Biological Evaluation

The biological behavior of the coatings was evaluated by bone marrow-derived mesenchymal stromal cells (BM-MSCs, ATCC PCS-500-012TM, Manassas, VA, USA). For the tests, samples were pre-wetted (1 mL of α-MEM was added to each sample), incubated at 37 °C with 95% humidity for 2 h, then 3 × 10^3^ BM-MSCs were seeded on each sample and maintained in culture for 14 days. Cell viability was monitored by Alamar Blue assay at 1, 3, 7 and 14 days. To exclude any potential toxic effect from material degradation by-products, the conditioned medium (CM) obtained from keeping the materials in α-MEM for 24 h was added to the previously seeded cell monolayer of BM-MSCs (5 × 10^3^) and maintained in culture for a further 24 h (37 °C; 95% humidity). Finally, the morphology of BM-MSCs seeded on the materials was made visible by staining actin filaments with phalloidin-fluorescein isothiocyanate (FITC) and nuclei with Hoechst 33258, at 3, 6 and 24 h after seeding to follow the steps of cell adhesion over time and compare the behavior of the different materials. To measure cell proliferation on the different coatings quantitatively, we measured the area covered by cells at 3 and 6 h, using the NIS-Element imaging software Advanced Research. The analysis was performed by light microscopy (Nikon, Tokyo, Japan) on five fields at 20× magnification.

## 3. Results and Discussion

### 3.1. Targets Characterization

**Figure 1:**
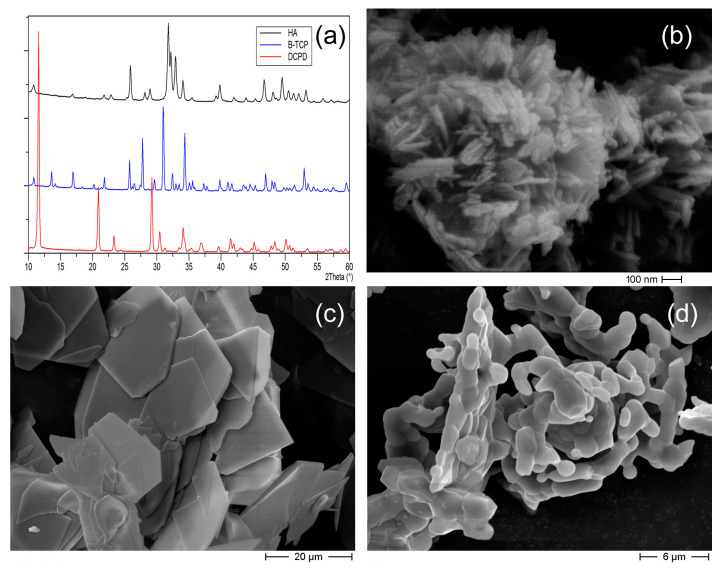
XRD patterns of the powder samples used for targets preparation (**a**); and SEM images of HA (**b**), DCPD (**c**) and β-TCP (**d**) particles.

The XRD patterns of HA, β-TCP and DCPD powders used for target preparation displayed intense and sharp peaks (Figure 1a), which showed the high crystallinity of these materials and matched the patterns of the pure phases reported in ICDD database (HA: PDF 9–432; β-TCP: PDF 9–169; DCPD 9–77). The morphologies of the different calcium phosphate particles were quite different. Indeed, HA was composed of elongated nanocrystals, whereas DCPD crystals displayed a plate-like morphology with sharp edges and large faces. β-TCP particles showed a typical morphology with rounded edges. Although β-TCP is very crystalline, no crystal faces or edges were appreciable because of the solid-state method of preparation (Figure 1).

### 3.2. Coating Characterization 

#### 3.2.1. Deposition of Different Calcium Phosphates

**Figure 2:**
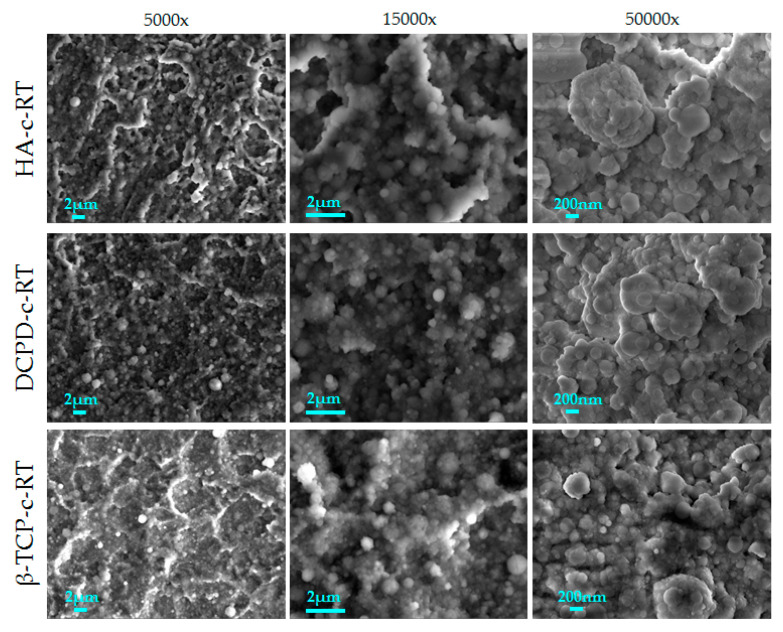
FEG-SEM images of the three different calcium phosphate coatings deposited at room temperature.

First, a comparison was carried out between the coatings obtained by the deposition of different calcium phosphates at room temperature. The morphology of the films is shown in Figure 2. All the coatings are uniform and homogenous and formed by nanosized globular aggregates. No defects were observed, such as cracks and delamination, or uncoated areas. Since the growth of the coatings was conformal, the surface finishing of the titanium alloy substrate at the microscale remained visible. At higher magnification (15,000× and 50,000×), the coatings revealed different dimension of aggregates, having diameters from ~80 nm (single aggregates) to 1.5 µm (clusters) (Table 1). In general, the average diameter was 250–300 nm for all samples. 

However, images at higher magnification (Figure 2, third column) showed some differences in the shape of the aggregates depending on the deposition target. Indeed, brushite showed a flatter morphology quite similar to that of hydroxyapatite, while β-TCP showed more spherical grains and had a higher dimensional homogeneity. This indicated that the target characteristics influenced the coating morphology. To show the differences between the coatings better, the minimum, maximum and average diameters were quantitatively measured (Table 1).

**Figure 3:**
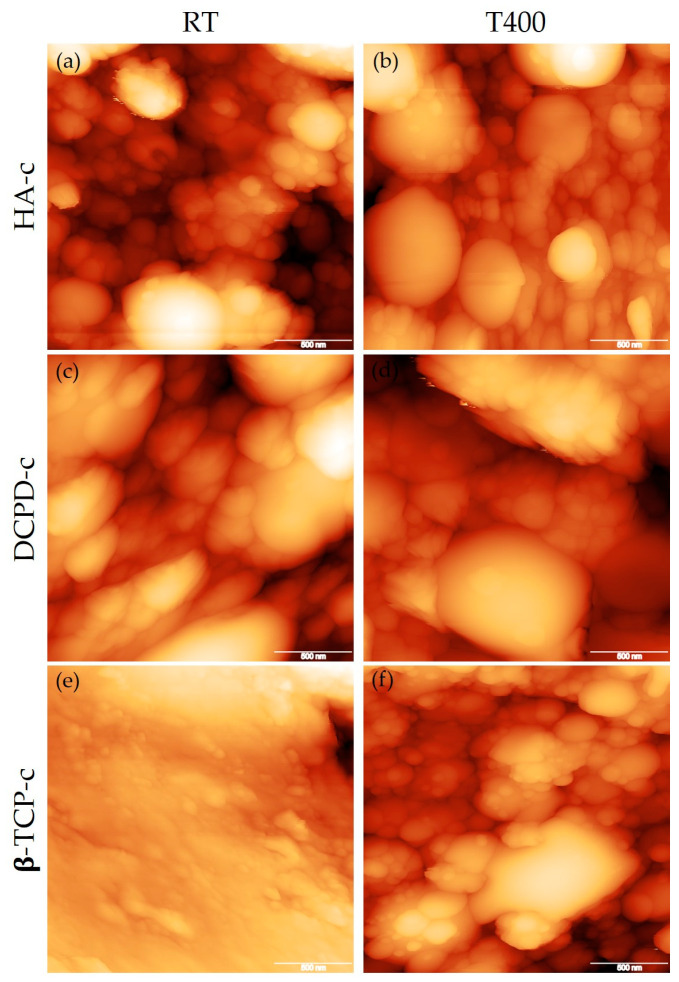
AFM images of HA-c (**a**), DCPD-c (**c**), β-TCP-c (**e**) deposited at room temperature and at 400 °C (**b**,**d**,**f**), respectively (scale bar: 500 nm).

Differences among the coatings were clearer when observing AFM images (Figure 3 and Table 2). Here, due to the characteristics of the deposition, all coatings showed high surface roughness. However, the value strongly varied among the three calcium phosphates, with hydroxyapatite and brushite showing similar values and β-TCP showing the lowest roughness. Film thickness also depended on the phosphate under examination with HA and DCPD exhibiting a higher thickness and β-TCP a lower one. We inferred that this behavior strongly depended on the mechanical characteristics of the targets obtained by the different materials and on their ability to retain their shape after uniaxial pressing, which depended on the powder size and shape. Indeed, β-TCP target was the least compact and more prone to experiencing some pulverization during deposition, which reduced the deposition rate.

**Figure 4:**
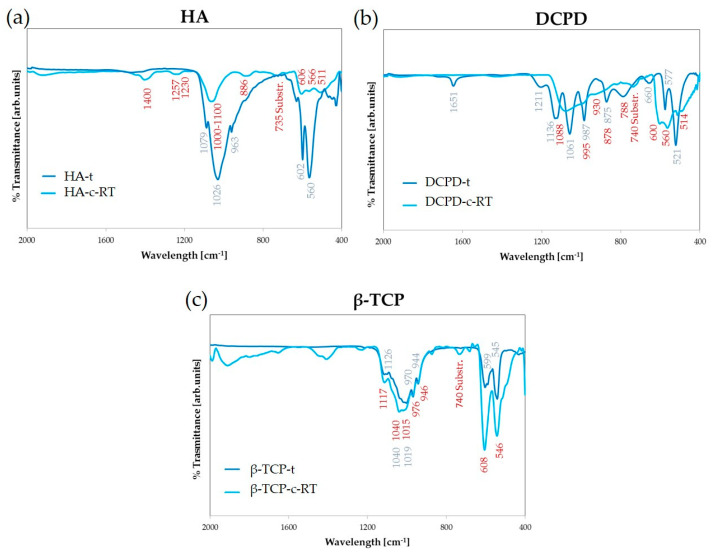
FT-IR spectrum of calcium-phosphates targets and room temperature coating (**a**): HA, (**b**): DCPD and (**c**): β-TCP.

Regarding composition, the FT-IR spectra of HA-c indicated that the coatings were composed of hydroxyapatite (Figure 4a). In fact, bands were observed at 1257 and 1230 cm^−1^ (HPO_4_ OH in-plane bend), 1100–1000 cm^−1^ (antisymmetric stretch ν_3_PO_4_), 606, 566 and 511 cm^−1^ (antisymmetric bend ν_4_PO_4_) [17]. In addition, bands were detected relating to the presence of carbonated ions around 1400 cm^−1^ (asymmetrical and symmetrical stretching modes of CO_3_ν_3_) and 886 cm^−1^ (ν_2_CO_3_) [17], which indicated that the deposition treatment induced carbonate ions in the structure of HA-c even if carbonate had been absent in the starting HA-t. In Figure 4b, the FT-IR spectra of DCPD-c displayed bands at 1088 cm^−1^ (antisymmetric stretch ν_3_PO_4_), 995 cm^−1^ (symmetric stretch ν_1_PO_4_), 600, 560 and 514 cm^−1^ (antisymmetric bend ν_4_PO_4_) [22]. In this case, there was a marked difference between the target and the coatings that could be observed in the bands in the 1200–870 cm^−1^ range, which is characteristic of phosphate stretching. Indeed, in the DCPD-t spectrum bands at 1211, 1136 and 1061 cm^−1^ (antisymmetric stretching ν_3_PO_4_) and 987 and 875 cm^−1^ (asymmetric stretch vibration P–O–P) were present, but only one band could be observed in the coating, indicating that the deposited phase was definitely amorphous. The FT-IR spectra of β-TCP-c (Figure 4c) showed bands at 1117 cm^−1^ (ν_3_HPO_4_ or combination), 1043 and 1015 cm^−1^ (antisymmetric stretch ν_3_PO_4_), 976 and 946 cm^−1^ (symmetric stretch ν_1_PO_4_), 608 and 546 cm^−1^ (antisymmetric bend ν_4_PO_4_) [15]. One of the advantages of the IJD technique is that it permits the preservation of the stoichiometry of the starting target in the corresponding coating, thereby avoiding the formation of decomposition phases or preferential sputtering, which are characteristic of other plasma-assisted techniques, such as plasma spray or magnetron sputtering [23]. Indeed, all bands in the spectrum here could be ascribed to CaP phases although all coatings deposited at room temperature were amorphous (Figures S1 and S2). In addition, we showed that the fidelity in stoichiometry conservation from the target to the coatings was phosphate-dependent since the β-TCP coating showed the highest similarity to the corresponding target, indicating a better stoichiometry transfer during deposition. 

EDS analyses (Table 3) indicate the presence of calcium and phosphorus in the coatings. From Table 3 it is possible to notice a correspondence between the Ca/P molar ratios of the targets and those of the coatings that confirms a correct stoichiometric transfer during deposition, as indicated also by FT-IR results. Data indicated that the similarity was at maximum for β-TCP and DCPD and was CaP powder phase-dependent. Indeed, HA coatings notably showed the lowest similarity to target composition. The Ca/P ratio for HA-c was similar to the Ca/P value obtained from other plasma-assisted deposition techniques: for example, pulsed laser deposition [9] and magnetron sputtering [23]. This also indicated that, in spite of being the most explored materials for plasma-assisted coating deposition, hydroxyapatite did not necessarily have the highest fidelity in composition transfer from target to coating in the IJD technique. 

#### 3.2.2. Deposition at High Temperature

**Figure 5:**
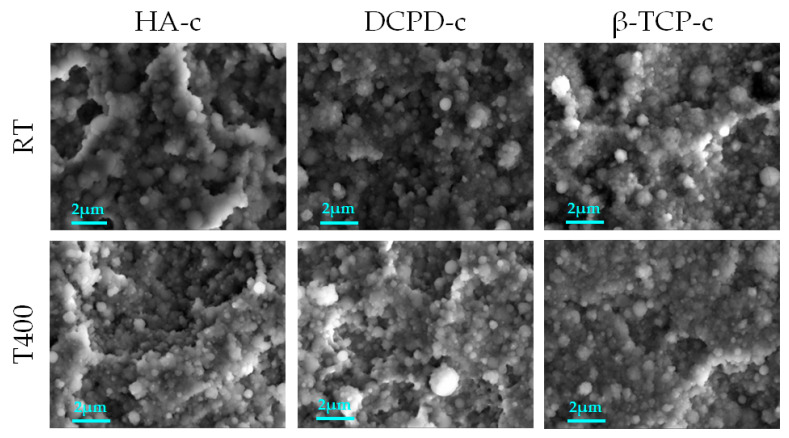
FEG-SEM images of room temperature coatings compared with films realized at 400 °C (15,000× magnification—scale bar: 2 µm).

The FEG-SEM images in Figure 5 directly compare the morphology of the films obtained at 400 °C and at room temperature. All coatings appeared uniform and showed homogeneous surface coverage irrespective of the phosphate or deposition temperature. No cracks, defects or signs of delamination were observed for any coating. In addition, no significant differences were assessed in the aggregate morphology, dimension or shape among the phosphates deposited at room temperature and those at 400 °C (Table 1), indicating that the morphology of the films was not significantly affected by heating, as was found in the literature for annealing after deposition [17].

However, the AFM results showed that heating had an impact on surface topography at a lower scale (Figure 3), resulting in an increase in surface roughness for DCPD and TCP but a slight reduction for HA. The same modifications were experienced in coating thickness: increases for DCPD and TCP and a decrease for HA. This phosphate-dependent response to deposition at high temperature had not been reported and needs to be taken into account when depositing a coating at different temperature. In general, an increase in surface roughness and in thickness in the absence of surface defects are considered beneficial, so deposition at high temperature appears promising. In fact, higher thickness, when in the submicrometric range, permits longer coating action and avoidance of the mechanical mismatch with the substrate that occurs for microscale coatings [3,24]. High surface roughness, instead, can promote cell adhesion and proliferation [25]. In addition, highly rough, multi-scale coatings, having nano- to micro-scale morphology were demonstrated to influence the differentiation of mesenchymal stem cells towards an osteogenic lineage positively, hence prospectively permitting faster osseointegration of the implants [11,25,26].

**Figure 6:**
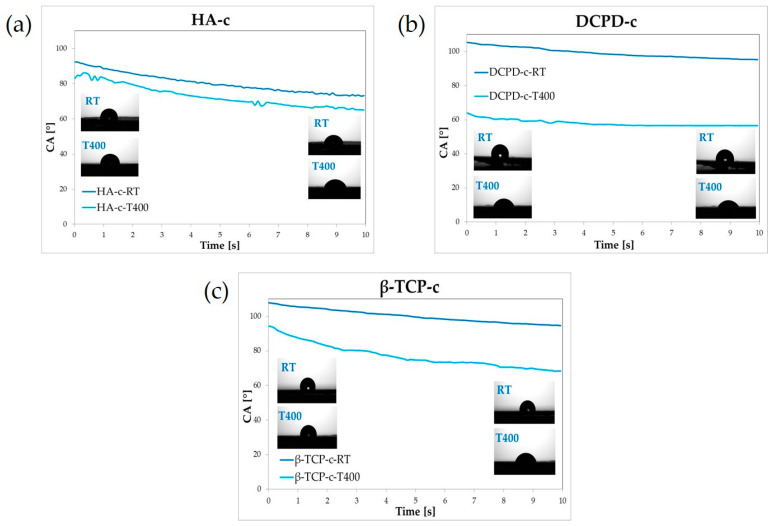
Wettability results for the different coatings realized (**a**) HA-c-RT and HA-c-T400, (**b**) DCPD-c-RT and DCPD-c-T400 and (**c**) β-TCP-c-RT and β-TCP-c-T400.

Wettability results are shown in Figure 6. While all films at 400 °C were hydrophilic, the DCPD and TCP films deposited at room temperature were slightly hydrophobic because as the contact angle was above 90°. In general, better hydrophilicity of the coating favors cell adhesion, differentiation and proliferation and bone mineralization, so a lower contact angle is generally desired [27]. 

Results showed that hydrophilicity can be improved by heating. Indeed, coatings realized at high temperature are more hydrophilic than their counterparts at room temperature since the contact angle decreases for all films, neglecting the CaP. This behavior had already been reported for post-treatment annealing of bovine apatite coatings [17]. The effect was more marked, with high temperature processing resulting in a switch from hydrophilic (contact angle 68°) to hyperhydrophilic coatings (5–15°). Wettability depend on a multitude of factors including surface chemistry, surface energy and topographical features [28]. Depending on the case, each of these elements can prevail. Our results indicated that the starting contact angle strongly depended on chemistry, i.e., on the calcium phosphate used for deposition (with HA being the roughest and most hydrophilic) and on its crystallinity. Hence, as observed for natural and synthetic ion-doped HA coatings deposited by IJD [14] at room temperature, surface chemistry prevailed over topography in determining the contact angle. After heating, however, surface roughness significantly increased and its contribution predominated over that of the surface chemistry. In any case, when compatible with the substrate, deposition at a higher temperature appeared more promising than deposition at room temperature, as it permitted higher hydrophilicity.

**Figure 7:**
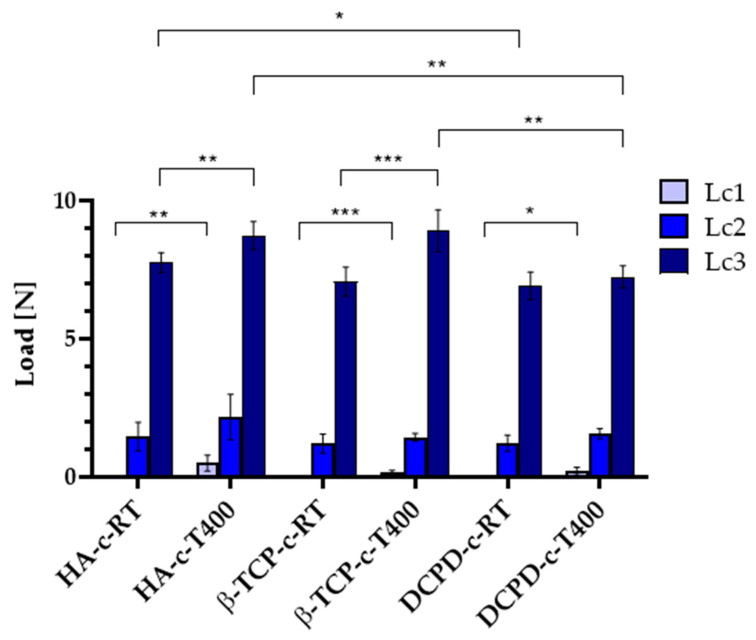
Results of scratch test. Critical failure loads for initial detachment (Lc1), delamination (Lc2) and complete penetration are shown for all samples. Only the *p* ≤ 0.05 was considered as statistically significant. (* *p*-value ≤ 0.05, ** *p* ≤ 0.01, *** *p* ≤ 0.001).

The results of the scratch test are reported in Figure 7. For all deposition conditions, coatings underwent failure and detachment as underscored by the presence of Lc3, but some differences were observed depending on the target material and deposition temperature. First, the absence of Lc1 was noticed in all coatings deposited at room temperature, which indicated an early detachment that occurs concurrently with the application of the load, revealing poor mechanical properties and scarce adhesion to the titanium substrate. Instead, when the coatings were deposited at 400 °C, we observed the presence of Lc1 and an increase in Lc3 for all CaPs, meaning that these coatings had higher adhesion compared to those deposited at room temperature. In fact, in case of HA-c, Lc3 increased from 7.75 ± 0.36 N at room temperature to 8.74 ± 0.51 N (*p*-value ≤ 0.01); for β-TCP-c, 7.07 ± 0.53 to 8.91 ± 0.76 N (*p*-value ≤ 0.001); and for DCPD-c, 6.92 ± 0.49 to 7.24 ± 0.40 N (*p*-value ≥ 0.05, no significant difference). These data indicated that the annealing treatment during deposition significantly increased the adhesion and resistance of the films to the starting (Lc1) and total (Lc3) detachment. The values obtained were higher than those for post-treatment annealing [17] and were in line with studies on HA films realized at different temperatures by pulsed laser deposition [29,30]. Indeed, the failure loads of 2–3 N reported in the literature were lower than the Lc3 values obtained in this research, indicating that the coatings obtained by IJD are promising for the proposed application. When comparing different materials, HA-c and β-TCP-c were found to be more adherent than DCPD-c, at both room temperature and 400 °C, but HA also showed the highest variability. 

Based on these results, deposition at 400 °C is preferred for all phosphates.

### 3.3. Stability Profile 

**Figure 8:**
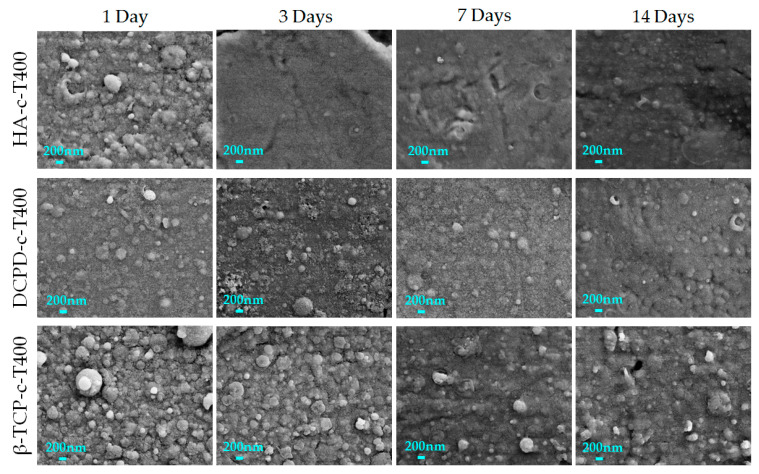
Morphology of the CaP coatings after 1 day (first column), 3 days (second column), 7 days (third column) and 14 days (last column) immersion in the medium.

The morphology of the coatings during dissolution tests was studied and is reported in Figure 8. FEG-SEM images of the coatings deposited at 400 °C (HA-c-400, β-TCP-c-400 and DCPD-c-400) showed how the films progressively dissolved but are still present on the titanium surface after 14 days immersion in α-MEM. In fact, although the films appeared progressively flatter and less thick, with a decrease in aggregates dimension and number, globular aggregates were still clearly visible at all timepoints, which indicated that the coatings were not completely dissolved. However, target-dependent differences are relevant. Indeed, for HA, significant dissolution occurred after the first 24 h, rendering the coating barely visible. DCPD showed the same dissolution pattern, but the coating remained thicker and more visible for up to 14 days. In β-TCP-c-400, the aggregates were more abundant and well defined, and showed low signs of dissolution even at the longer timepoints, indicating a higher stability. Importantly, although no signs of delamination were detected, some isolated cracks were observed, especially for HA (Figure S3), where they started to form already at 3 days. Some isolated cracks were also noticed for brushite, but they started to form at 14 days, meaning they could have been ascribed to the cracks in titanium substrate, which was initially concealed by the higher thickness of the coating.

Data obtained for films stability did not correlate with the solubility of the starting calcium phosphate targets because, in our case, all the phosphate-based coatings were highly amorphous even when deposited at 400 °C. As a consequence, the solubility of the coatings was not dominated by the starting materials but by the coatings’ surface roughness and the formation of cracks and detachments. These in turn depended on coating adhesion and the possible presence or absence of a soluble phase in the coating.

### 3.4. Biological Evaluation 

**Figure 9:**
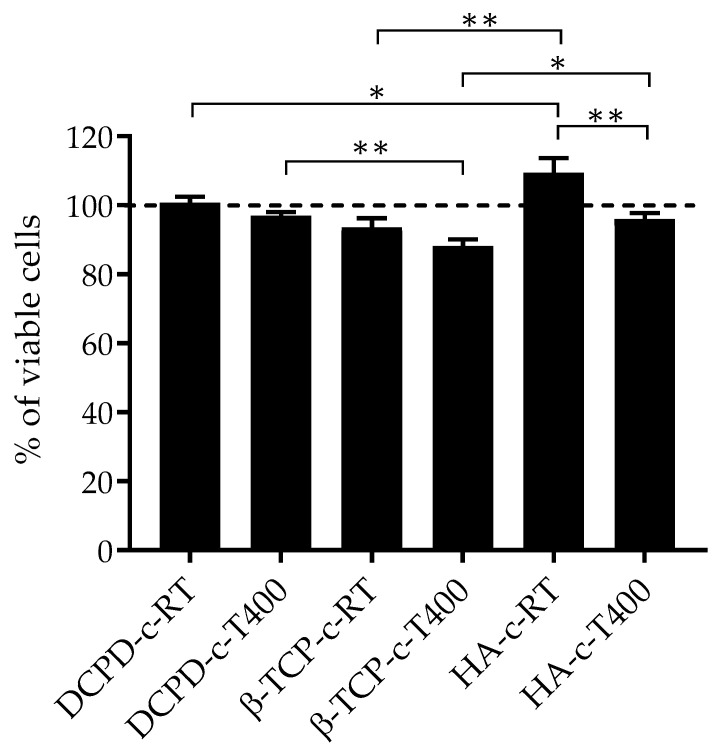
Cell viability by the Alamar Blue assay applied to the BM-MSC monostrate maintained in culture with CM for 24 h. The dashed line represents the CTRL culture that had 100% cell viability. Only *p* ≤ 0.05 was considered as statistically significant (* *p*-value ≤ 0.05, ** *p* ≤ 0.01).

No significant cytotoxic effects were detected when BM-MSCs were cultured with CM for 24 h, confirming that the degradation by-products released from the coatings are not toxic for cells, as reported in Figure 9. 

**Figure 10:**
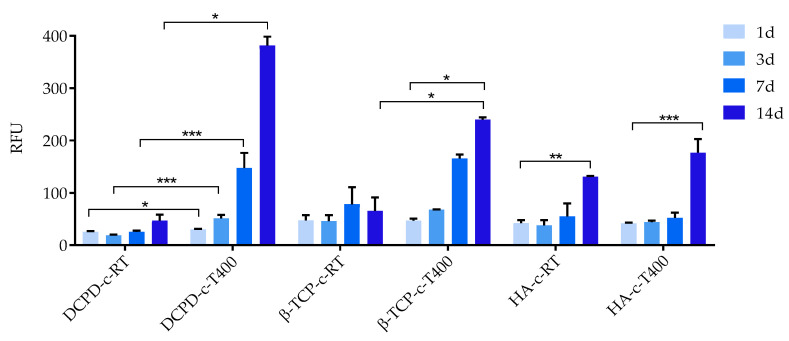
Cell viability by Alamar Blue assay applied to BM-MSCs seeded on materials and maintained in culture for 14 days. Only the *p* ≤ 0.05 was considered as statistically significant (* *p* ≤ 0.05, ** *p* ≤ 0.01, *** *p* ≤ 0.001).

The results of the cell viability of the BM-MSCs seeded on materials and maintained in culture for 14 days are shown in Figure 10. The results of Alamar Blue assay confirmed a good viability of BM-MSCs for all materials at all the considered timepoints. Figure 10 clearly shows relevant cell growth over time for all materials with coating treated at 400 °C and to a lesser degree for HA-c-RT.

In general, comparing the coatings obtained by the different phosphates deposited at room temperature, HA-c-RT showed the best behavior. For the other CaP coatings, especially for DCPD-c-RT, viability seemed quite scarce, indicating excessive solubility of the coatings leading to an overly high ion release [31]. 

All coatings deposited at high temperature showed better biological behavior compared to those deposited at room temperature. Indeed, cells viability values significantly increased for all phosphates, especially at longer timepoints. Interestingly, in this case, the best behavior was shown by DCPD-c-T400 and β-TCP-c-T400, while the performance of HA-c-T400 was poorer and not much different from the behavior of the coating deposited at room temperature. It could be inferred that this behavior depended on the formation of cracks and on delamination resulting from the formation of more soluble regions and from their dissolution (FEG-SEM, Figure 9). This behavior had been observed in a study that investigated hydroxyapatite coatings for dental applications [32]. There, dental pulp stem cells were cultured on HA coatings deposited at room temperature and annealed after deposition. The coatings were observed by digital image correlation at different timepoints of cells culturing. The results indicated that HA coatings deposited at room temperature experienced excessive and overly fast dissolution, while delamination was evident in coatings after annealing. Both behaviors led to poor cell viability. In the present study, we used a different cell source, but we found the same behavior, indicating that this was dependent on the characteristics of the coatings, not of the cells. In addition, no significant differences were observed in the behavior of the coatings when deposition at high temperature was performed instead of post-treatment annealing. Data obtained here seemed to contrast with those of the conditioned medium. However, this was not the case, as the detrimental effect of HA coatings on cell viability had to be ascribed to delamination, which hampered cell adhesion and film proliferation, rather than to a toxic effect of the material itself. Importantly, these results showed that, in spite of being the most investigated of the phosphates, brushite had the highest cell viability. HA, being the most used, had the lowest performance. As a consequence, more research is needed to investigate brushite-based coatings in vitro and in vivo. 

**Figure 11:**
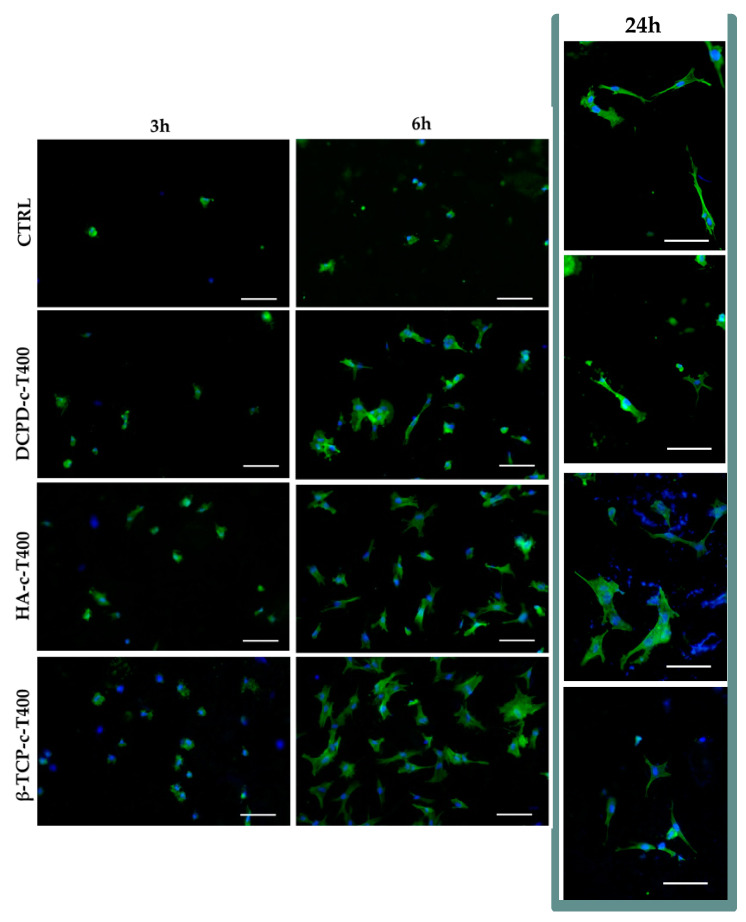
Representative images of BM-MSCs stained with phalloidin-FITC (green) and Hoechst 33258 (blue) to marked actin filaments and nuclei, respectively. Scale bars 100 µm.

Figure 11 shows the adhesion of BM-MSCs on CTRL (bare metallic substrate) and on HA-c-T400, β-TCP-c-T400, DCPD-c-T400 coatings at 3, 6 and 24 h. The early steps of cell adhesion were considered for observing potential differences in cell morphology during adhesion to the nanostructured coatings. 

**Figure 12:**
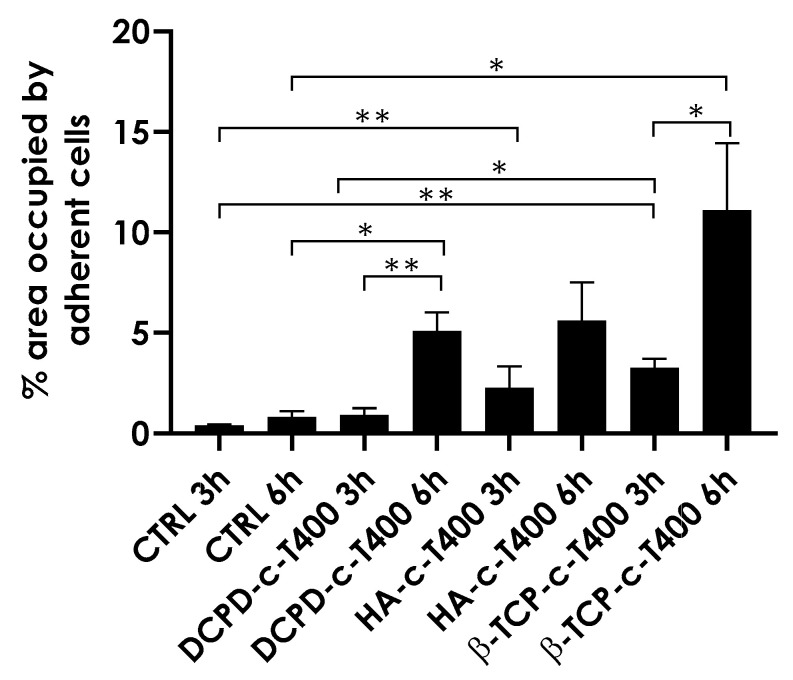
Quantification of the area occupied by BM-MSCs that adhered to different coating surfaces, expressed as percentage of the total area. The quantification is related to 3 h and 6 h timepoints (* *p*-value ≤ 0.05, ** *p* ≤ 0.01).

Overall, CTRL and all three coatings supported cell adhesion as demonstrated by the presence of adherent cells at 3 h. All coatings showed increased cell proliferation at very early timepoints (3 and 6 h), suggesting that nanostructuring promoted better cell attachment and spreading. Importantly, to mimic the gold standard in orthopedic implants, we selected micro-rough Ti6Al4V discs as control to measure the ability of our coatings to increase cell adhesion and proliferation compared to the clinically available references. To evaluate early cell adhesion better, we quantified the percentage of the area covered by the adherent cells that were analyzed (Figure 12). The results showed that cell coverage, indicative of cells proliferation, was higher for β-TCP-c-T400 compared to HA-c-T400 and DCPD-c-T400 at both 3 and 6 h.

Some important differences in cells morphology could also be appreciated (Figure 11). Indeed, at 3 h, BM-MSCs cultured on the coatings had a slightly more stretched morphology compared to CTRL. The difference increased notably at 6 h as observed by cell spreading on coatings, especially on β-TCP-c-T400 compared to CTRL. At 24 h, BM-MSCs had a fibroblast-like shape on CTRL and DCPD-c-T400, while they showed a more flattened and branched shape on HA-c-T400 and β-TCP-c-T400. This effect on cell morphology, spreading and branching is important because, as we previously observed, this was an index of early differentiation towards an osteogenic lineage in nanostructured bone apatite coatings deposited on different substrates [11,19].

For this reason, both DCPD coatings, which promoted greater cell proliferation, and β-TCP, which favored a branched morphology, are promising and worth further characterization.

## 4. Conclusions

For the first time, deposition of different calcium phosphates (HA, DCPD and β-TCP) by ionized jet deposition at room temperature and 400 °C were systematically compared.

The results showed that IJD obtains tunable coatings, the composition of which resembles that of the deposition target. All coatings were nanostructured comprising grains of about 80 nm in aggregate clusters up to 1.5 µm. Small differences were noticed in the shape and uniformity of the grains, and significant difference were assessed in the coating surface roughness depending on the phosphate.

Upon heating, coating adhesion to the substrate and hydrophilicity improved as did stability in a calcium phosphate-dependent fashion. Indeed, upon exposure to a medium, all coatings deposited at room temperature showed excessive dissolution, leading to low cell viability. All coatings deposited at 400 °C progressively dissolved, but the three phosphates showed different stability and mode of dissolution, with β-TCP being the most stable and hydroxyapatite the most susceptible to cracking during dissolution. As a result, hydroxyapatite also had the lowest cell viability at the early and late timepoints. In contrast, brushite coatings promoted the best cell viability of the three coatings.

When observing the morphology of cells cultured on the coatings, all three supported cell adhesion and proliferation at 3 h. In addition, the different phosphates had a different impact on cell morphology. Indeed, at 6 h cell spreading for all coatings was higher compared to control. At 24 h, BM-MSCs cultured on β-TCP showed a greater spread and branched morphology, which was a sign for early differentiation towards an osteogenic lineage. 

These results showed that different calcium phosphate phases can be deposited by IJD and that the use of different starting materials is a route to tune the physicochemical, morphological, mechanical and biological characteristics of the coatings.

## Figures and Tables

**Table 1:** Aggregate size distribution measured by SEM (Dm: maximum diameter, dm: minimum diameter and da: average diameter).

	Dm	dm	da	Size Distribution (% of Aggregates for Each Range)				
	(nm)	(nm)	(nm)	d < 100 nm	100 nm ≤ d < 250 nm	250 nm ≤ d < 500 nm	500 nm ≤ d < 1000 nm	d ≥ 1000
HA-c-RT	1372 ± 221	79 ± 25	304 ± 209	1	53	35	9	3
HA-c-T400	1045 ± 161	106 ± 12	296 ± 174	1	53	36	10	1
DCPD-c-RT	1293 ± 684	105 ± 3	307 ± 221	0	49	41	8	1
DCPD-c-T400	1550 ± 379	106 ± 13	296 ± 194	1	48	43	6	2
β-TCP-c-RT	1526 ± 203	118 ± 9	278 ± 212	0	63	31	4	2
β-TCP-c-T400	1530 ± 345	105 ± 9	262 ± 208	1	67	25	5	2

**Table 2:** Values of RMS and maximum coating height measured by AFM.

	RMS (nm)		Maximum Height (nm)	
	RT	T400	RT	T400
HA-c	107	62	505	423
DCPD-c	98	130	537	661
β-TCP-c	26	96	238	507

**Table 3:** Composition of the different coatings, as assessed by EDS.

Ca/P	HA	DCPD	β-TCP
Stoichiometric (target)	1.67	1	1.50
Coating	1.91	0.83	1.42

## Data Availability

The data presented in this study are available on request from the corresponding authors.

## Supplementary Materials

The following supporting information can be downloaded at: https://www.mdpi.com/article/10.3390/nano13111758/s1, Figure S1: XRD pattern of HA-c-T400; Figure S2: XRD pattern of coatings collected for longer collection times in the relevant range of 2θ degrees; Figure S3: Morphology of the CaP coatings before treatment, and after 1, 3, 7 and 14 days immersion in medium.
