# Supplementary material for: Ionized Jet Deposition of Calcium Phosphates-Based Nanocoatings: Tuning Coating Properties and Cell Behavior by Target Composition and Substrate Heating
Source: Nanomaterials (Basel). 2023 May 29;13(11):1758. doi: 10.3390/nano13111758 (PMC10254152; doi:10.3390/nano13111758)
Supplement: Supplementary file 1 [file nanomaterials-13-01758-s001.zip › nanomaterials-2407823-supplementary.pdf]

Supplementary Material

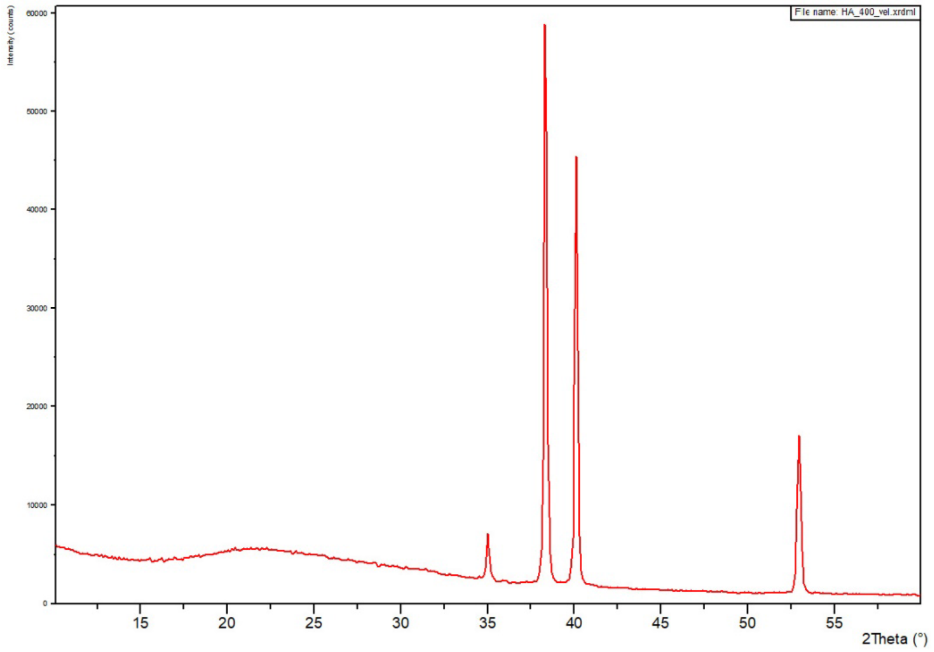

Figure S1 – XRD pattern of HA-c-T400. The peaks are attributed to Ti from the metallic substrate (ICDD card# 01-089-3725)

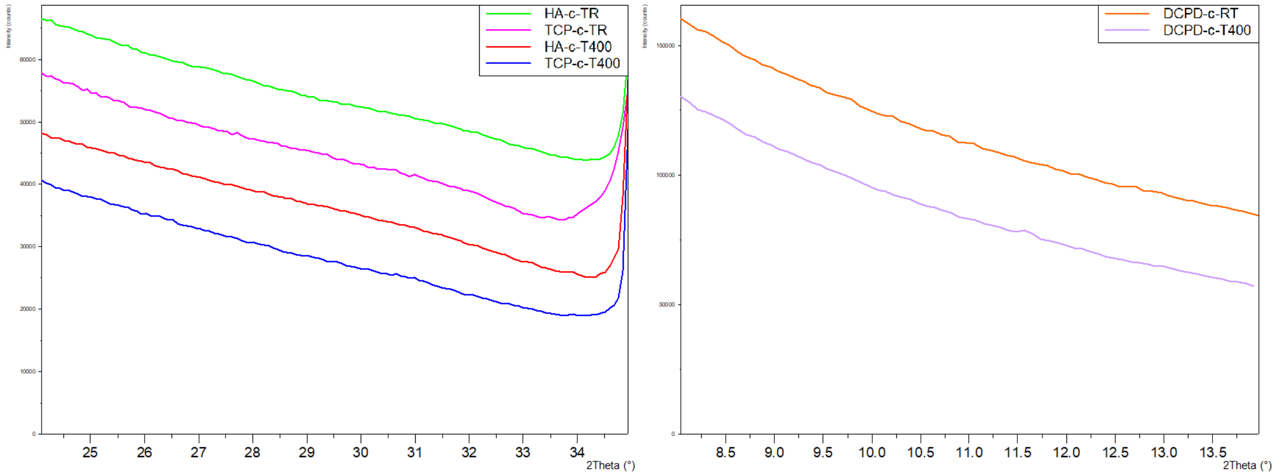

Figure S2 – XRD pattern of coatings collected for longer collection times in the relevant range of 2θ degrees.

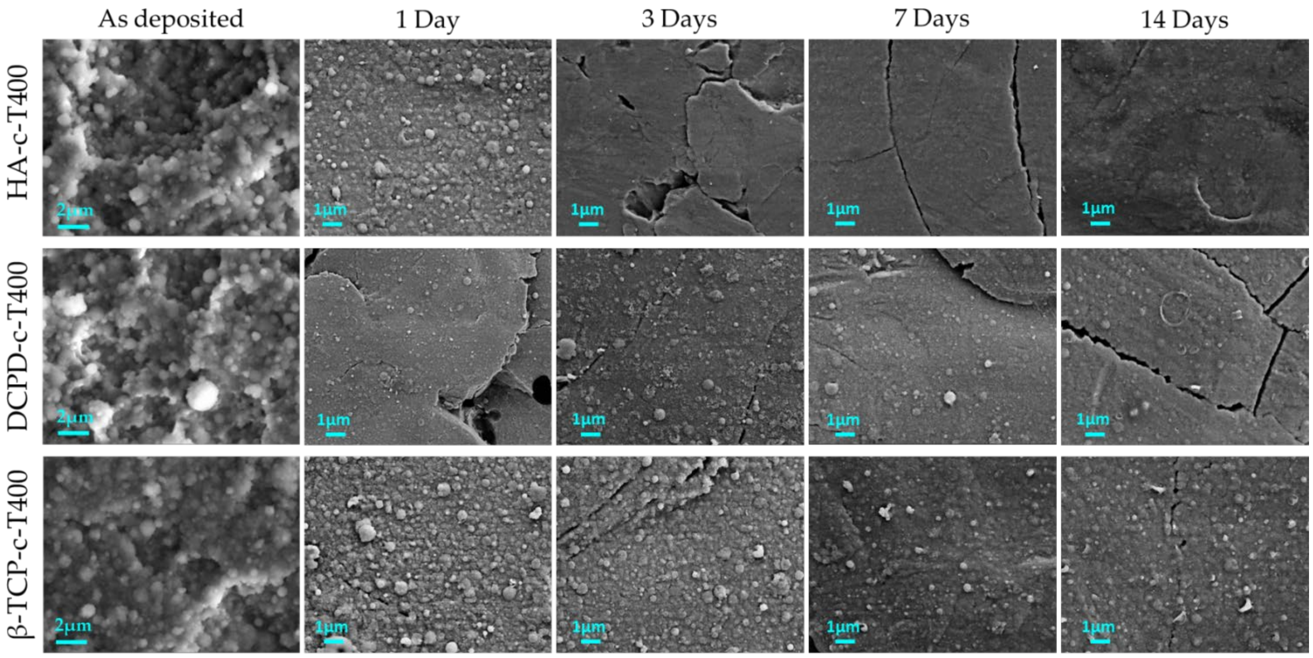

Figure S3 - Morphology of the CaP coatings before treatment (first column), and after 1 (second column), 3 (third column), 7 (fourth column) and 14 days (last column) immersion in medium.
